# Rumen Cellulosomics: Divergent Fiber-Degrading Strategies Revealed by Comparative Genome-Wide Analysis of Six Ruminococcal Strains

**DOI:** 10.1371/journal.pone.0099221

**Published:** 2014-07-03

**Authors:** Bareket Dassa, Ilya Borovok, Vered Ruimy-Israeli, Raphael Lamed, Harry J. Flint, Sylvia H. Duncan, Bernard Henrissat, Pedro Coutinho, Mark Morrison, Pascale Mosoni, Carl J. Yeoman, Bryan A. White, Edward A. Bayer

**Affiliations:** 1 Department of Biological Chemistry, The Weizmann Institute of Science, Rehovot, Israel; 2 Department of Molecular Microbiology and Biotechnology, Tel Aviv University, Ramat Aviv, Israel; 3 Microbial Ecology Group, Rowett Institute of Nutrition and Health, University of Aberdeen, Aberdeen, United Kingdom; 4 Architecture et Fonction des Macromolecules Biologiques, Aix-Marseille University and Centre National de la Recherche Scientifique (CNRS), Marseille, France; 5 University of Queensland Diamantina Institute, Woolloongabba, Queensland, Australia; 6 Department of Animal Sciences, The Ohio State University, Columbus, Ohio, United States of America; 7 The French National Institute for Agricultural Research (INRA), UR454 Unité de Microbiologie, Saint-Genès-Champanelle, France; 8 Department of Animal and Range Sciences, Montana State University, Bozeman, Montana, United States of America; 9 The Institute for Genomic Biology, University of Illinois, Urbana, Illinois, United States of America; 10 Department of Animal Sciences, University of Illinois, Urbana, Illinois, United States of America; INRA Clermont-Ferrand Research Center, France

## Abstract

**Background:**

A complex community of microorganisms is responsible for efficient plant cell wall digestion by many herbivores, notably the ruminants. Understanding the different fibrolytic mechanisms utilized by these bacteria has been of great interest in agricultural and technological fields, reinforced more recently by current efforts to convert cellulosic biomass to biofuels.

**Methodology/Principal Findings:**

Here, we have used a bioinformatics-based approach to explore the cellulosome-related components of six genomes from two of the primary fiber-degrading bacteria in the rumen: *Ruminococcus flavefaciens* (strains FD-1, 007c and 17) and *Ruminococcus albus* (strains 7, 8 and SY3). The genomes of two of these strains are reported for the first time herein. The data reveal that the three *R. flavefaciens* strains encode for an elaborate reservoir of cohesin- and dockerin-containing proteins, whereas the three *R. albus* strains are cohesin-deficient and encode mainly dockerins and a unique family of cell-anchoring carbohydrate-binding modules (family 37).

**Conclusions/Significance:**

Our comparative genome-wide analysis pinpoints rare and novel strain-specific protein architectures and provides an exhaustive profile of their numerous lignocellulose-degrading enzymes. This work provides blueprints of the divergent cellulolytic systems in these two prominent fibrolytic rumen bacterial species, each of which reflects a distinct mechanistic model for efficient degradation of cellulosic biomass.

## Introduction

The bovine rumen hosts a wide range of strictly anaerobic and some facultatively anaerobic microorganisms [1]–[5]. The rumen microbiota is highly diverse, including both prokaryotic and eukaryotic anaerobes, that maintains a mutualistic relationship with its host [6]. On the one hand, the rumen flora is dynamic and known to adapt to changes in the host diet and age [7], [8]. On the other, the rumen microbiota produces large quantities of short-chain fatty acids that are absorbed across the rumen wall and used as energy sources by the host [9]. Fermentation of plant material by rumen fiber-degrading microorganisms in the rumen typically provides 70% of the energy obtained from the diet [10]. Herbivore health and productivity are greatly affected by the composition and activity of the rumen microbiota and, in particular, by fiber-degrading species. Relatively few rumen bacteria have been identified as primary degraders of plant fiber, but cellulolytic *Ruminococcus* and *Fibrobacter* species clearly play an important role [11], [12]. Knowledge of the fibrolytic mechanisms employed by these specific rumen bacteria is of great importance for manipulation of animal diet and for improvement of its performance. Moreover, insights in this field may lead to biotechnological applications related to biofuel production.

Two cellulolytic Firmicutes bacteria, *Ruminococcus flavefaciens* and *Ruminococcus albus*, and the gram-negative *Fibrobacter succinogenes* are important and culturable cellulose-degrading agents in the rumen [2]. These three species are able to adhere and grow on cellulosic polysaccharides as their primary carbon and energy sources and in doing so breakdown plant cell wall material [13].

Efficient degradation of plant cell-wall polysaccharides by some anaerobic bacteria is achieved by a multienzyme complex specialized in cellulose degradation, known as the cellulosome, which has been best studied in *Clostridium thermocellum* [14]–[19]. The cellulosome is a molecular platform that assembles a multiplicity of carbohydrate-degrading enzymes, i.e., glycoside hydrolases (GHs), polysaccharide lyases (PLs) and carbohydrate esterases (CEs). These are degradative enzymes, such as endoglucanases, cellobiohydrolases, xylanases, etc., which attack heterogeneous, insoluble cellulosic substrates in a synergistic manner [18], [20]–[22]. Unlike other (notably aerobic) bacteria and fungi, these enzymes are not freely diffusible, because they contain a dockerin module that mediates their integration into the major cellulosome structural subunits, termed scaffoldins. The dockerin strongly interacts with multiple copies of cohesin modules located on the scaffoldins via a high-affinity protein-protein interaction [23]–[27]. In *C. thermocellum*, the scaffoldin also contains a carbohydrate-binding module (CBM) that binds the cellulosome complex to the plant cell wall substrate [28]–[31]. Thus, dockerin-containing enzymes are incorporated into scaffoldin-borne cohesins, and a CBM-bearing scaffoldin targets the assembly to the carbohydrate substrate. Moreover, the *C. thermocellum* cellulosomes are attached to the bacterial cell surface by virtue of an S-layer homology (SLH) domain [32].

One of the most elaborate cellulosomal architectures was recently discovered in *R. flavefaciens* through extensive study of its genome sequence and transcriptome [33], [34]. *R. flavefaciens* codes for more than a dozen cohesin-containing proteins that may interact with an unprecedented number (∼220) of dockerin-containing proteins. These early studies on the cellulosome of this bacterium established new features that deviate from those of the canonical *C. thermocellum* cellulosome. In *R. flavefaciens*, the ScaC protein bears both a cohesin and a dockerin module and serves as an “adaptor” scaffoldin [35]. Additionally, the cellulosome is attached to the bacterial cell surface in an unconventional manner, whereby a singular type of scaffoldin, ScaE, is covalently fastened to the cell-wall envelope via proteolytic cleavage and transfer by sortase-mediated attachment [36]. Previous analysis of *R. flavefaciens* dockerins [34] has served to classify the dockerins into at least six major groups, according to their conserved sequence profiles, and demonstrated the modular nature of the enzymes and their association to the other non-catalytic proteins. The characteristics of the cohesin-containing proteins and additional elements have yet to be described in detail.

In contrast to the elaborate cellulosome evident in *R. flavefaciens*, the system of *R. albus* remains puzzling. Despite the fact that *R. albus* produces an array of dockerin-bearing proteins [37], no genes encoding cohesin-containing proteins have been determined, and the presence of a defined cellulosome is thus in question. In previous work, several of its dockerin-containing endoglucanases were indeed characterized [38], [39]. *R. albus* is also known to adhere tightly to cellulose and appears to utilize several types of cellulose-adhesion mechanisms for this purpose, such as Pil proteins [40]–[43] and an exopolysaccharide glycocalyx [44]–[47]. Surprisingly, the major Cel48 exoglucanase that commonly characterizes cellulosomes in other bacterial species was found to bear a distinctive type of CBM rather than a dockerin at its C terminus [48]. This family 37 CBM was found to bind to numerous types of polysaccharides and was identified in several enzymes with catalytic modules such as GHs, PLs and CEs [49], [50]. Subsequent studies indicated that *R. albus* utilizes CBM37s to mediate bacterial cell surface attachment [51]. Moreover, CBM37 was shown to be exposed at the cell surface of *R. albus* 20 by Rakotoarivonina [50], who proposed that the adhesion and fibrolytic systems of *R. albus* are linked.

The recent availability of genomic data of *R. flavefaciens* and *R. albus* strains has enabled us to unravel the blueprint of the cellulolytic systems of ruminococci and to compare their alternative fiber-degrading strategies. Comparative genome-wide analysis has allowed the identification of structural elements of each cellulosome, such as scaffoldins and CBMs, and to assess the profile of dockerin-containing proteins and carbohydrate-degrading enzymes in each strain. This work provides a framework for the cellulose-degrading systems of these two ruminococcal species, thereby demonstrating both core elements and novel strain-specific enzymes, which would either assemble into a multi-enzyme cellulosome or comprise an array of cell-bound carbohydrate-active enzymes and associated proteins for *R. flavefaciens* and *R. albus*, respectively.

## Results

### Six available *Ruminococcus* genomes

**Figure 1:**
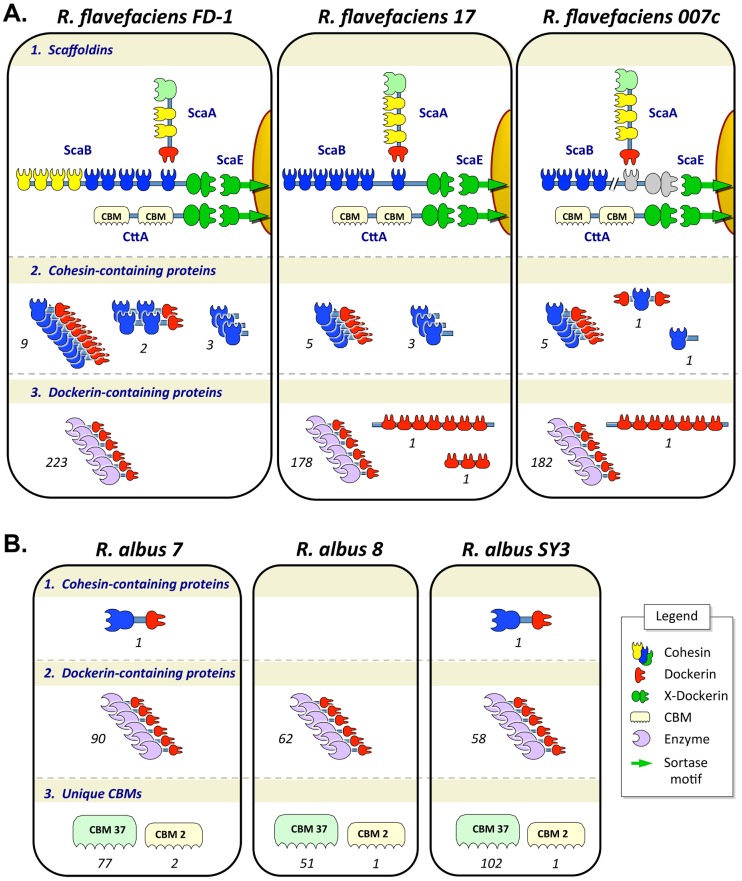
Blueprints of the cellulosome-related proteins in the designated strains of (A) *R. flavefaciens* and (B) *R. albus*, studied in this work. Schematic representation of scaffoldins, cohesin- and dockerin-containing proteins, which were identified in the genomes of each strain in this work. Numbers indicated the copy number of each type of protein architecture, identified in the designated strain. Legend of pictograms is shown in Panel B. See text for details.

The ability of cellulolytic bacteria to degrade plant cell-wall carbohydrates is encoded in their genomes. In this work, we explored the genomes of three strains each of *Ruminococcus flavefaciens* (FD-1, 17 and 007c) and *Ruminococcus albus* (7, 8 and SY3). Using a comparative bioinformatics approach, we identified their putative cellulolytic enzymes and, particularly for these two ruminococcal species, their cellulosome-related components (Fig. 1 and Table 1). Two new genomes, *R. flavefaciens* 007c and *R. albus* SY3, were sequenced and submitted to GenBank (see relevant sections in Materials and Methods). Although each of the six genomes was derived from bacteria obtained from a different cow and isolated at different geographical locations and time periods, it has been established that various species and strains coexist at the same time in the rumen of a given host organism [52], [53]. In an attempt to profile the cellulose-degrading strategy of each bacterium, each genome was examined in this work to identify homologs of the primary building blocks of the cellulosome, namely cohesin-containing proteins and dockerin-containing proteins, together with CBMs. We further applied various sequence analysis methods to identify and analyze the presence of known carbohydrate-active enzymes (CAZymes, [54], i.e., GHs, PLs and CEs) as detailed below. The following analyses were based on draft genome sequences (except for *R. albus* 7), showing an adequate level of genome coverage (see Materials and Methods), yet may include sequence gaps which restrict some of the information.

**Table 1:** Overview of key cellulosomal components identified in this work.

Cellulosome-producing bacteria	Cohesin-containing proteins	Cohesin modules	Dockerin-containing proteins	CAZymes			Multifunctional proteins		CBMs	
				GHs	PLs	CEs	Total	Novel^a^	Total	CBM37
*R. flavefaciens* FD-1	17	27	223	107	17	30	23	14	63	-
*R. flavefaciens* 17	11	21	180	96	4	23	19	2	52	-
*R. flavefaciens* 007c	10	16	183	95	4	23	17	-	51	-
*R. albus* 7	1	1	90	97	7	18	8	-	129	77
*R. albus* 8	-	-	62	90	6	18	4	4	88	51
*R. albus* SY3	1	1	58	104	4	16	7	1	155	102

a Strain-specific proteins, see Tables 4 and 5.

### Multiple architectures of cohesin-bearing scaffoldins in *R. flavefaciens* strains

We identified numerous cohesin-containing proteins in all three *R. flavefaciens* strains. Specifically, 17, 11 and 10 scaffoldin subunits were detected in strains FD-1, 17 and 007c, respectively (Table 1 and Fig. 1A). *R. flavefaciens* cellulosomes contain a unique spectrum of type-III cohesin modules [36], [55], [56], which are different than the type-I and type-II cohesins found in *C. thermocellum* and other cellulosome-producing clostridia. Type-III cohesin-containing proteins can be further catalogued into four functional groups according to their architecture:

- As demonstrated in earlier publications for strains 17 and FD-1, ScaA and ScaB serve as major scaffoldin subunits with multiple non-identical repeats of cohesin modules (Fig. 1A.1). ScaA harbors a unique type of C-terminal dockerin and ScaB contains a C-terminal X-dockerin (XDoc) modular dyad [56]. Notably, the composition of the major cohesins in the ScaB scaffoldin is different between the FD-1 strain (which contains two subtypes of cohesins on the same scaffoldin) and the 17 strain (in which all cohesins are of the same subtype) [57]. In addition, the number of cohesin repeats in ScaB varies between the *R. flavefaciens* strains, whereby strain 17 contains 7 cohesin repeats and strain FD-1 contains 9 repeats. ScaB of strain 007c contains at least 4 cohesins, but since its ORF (EWM54563) is located near the end of a contig in the draft genome, its C-terminus sequence is incomplete by definition (no stop codon was observed). Moreover, the presence of an XDoc modular pair in this strain can thus not be verified at this time. Yet it is clear that its sequenced cohesins are of the ScaA variety that resemble those of strain 17 as opposed to cohesins 1–4 of the FD-1 ScaB. We therefore presume that the 007c ScaB bears a single subtype of cohesin, the exact number of which is currently unknown.
- ScaE-like proteins (Fig. 1A.1) were identified in all three genomes. As shown for strains 17 and FD-1 in previous works, this type of scaffoldin has an important anchoring function, due to its ability to anchor the ScaB and CttA proteins [58] and to the presence of a C-terminal sortase sequence, which is involved in the attachment of the cellulosome to the bacterial cell surface [36]. In turn, CttA attaches to cellulose through its two CBMs, and the bacterial cell itself is thus attached to the substrate through this mechanism [58].
- The current work has revealed a third group of proteins (5–11 copies, according to the strain), characterized by a bi-modular theme, which includes both a single cohesin module and a single dockerin in the same polypeptide (Fig. 1A.2). As shown previously for ScaC in strain 17 [35], this type of protein may serve as an adaptor protein to regulate binding of either particular scaffoldins and/or enzymes into cellulosome complexes, thereby altering the repertoire of cellulosome content. Interestingly, this study indicates that *R. flavefaciens* FD-1 exclusively contains a second potential variation of this theme, in the form of two proteins that bear a C-terminal dockerin with two cohesins instead of one.
- In addition, we identified several scaffoldins (1–3 copies per strain) in the present research that bear a single cohesin module, which is >90% similar between strains 17 and 007c and ∼60% similar between strains FD-1 and 007c. These cohesins lack a dockerin module but are fused to a protein region whose function is as yet unknown (Fig. 1A.2).

**Figure 2:**
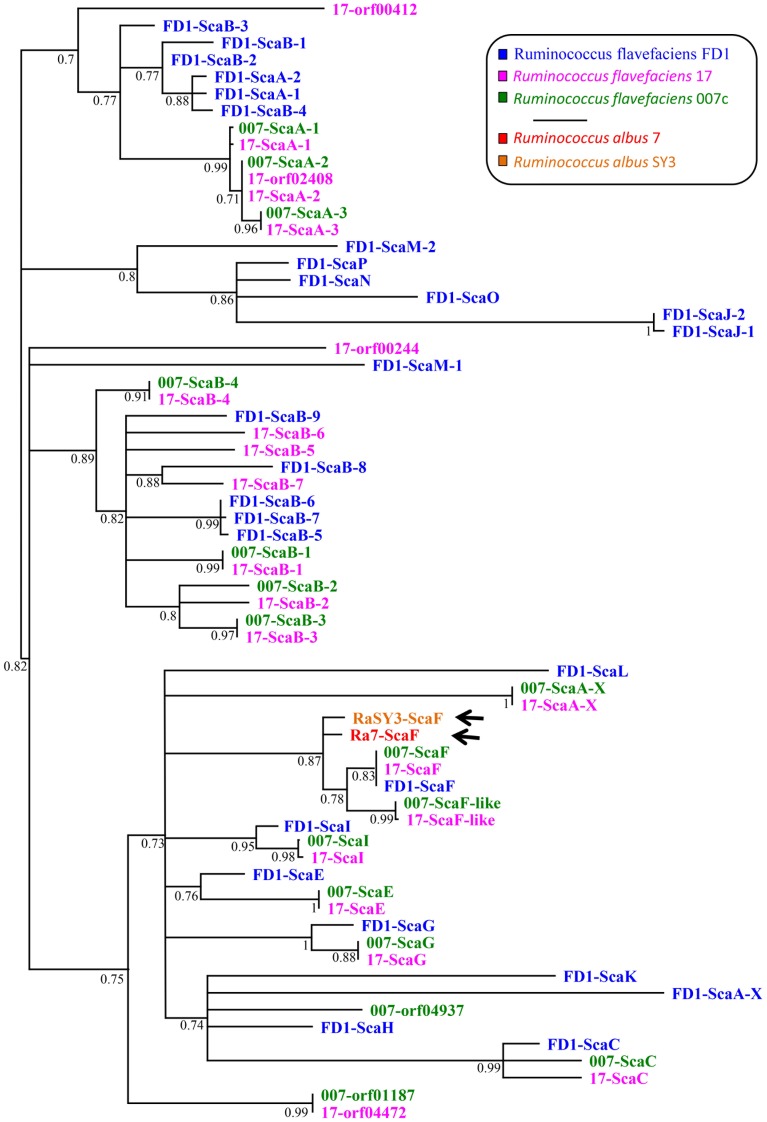
Phylogenetic relationship among cohesin modules of *R. flavefaciens* and *R. albus*. The names of the different cohesins are color coded according to the given strains. The various cohesins from the different strains were named based on the sequence similarity to those of the *R. flavefaciens* FD-1 strain (Table S1). The single cohesins identified in the two *R. albus* strains (arrows) cluster with those of the ScaF cohesins of *R. flavefaciens* and were hence labeled ScaF. Branches with bootstrap values below confidence level 0.7 were collapsed.

In order to evaluate the sequence relatedness among the cohesins from the different *R. flavefaciens* strains, we constructed a phylogenetic tree (Fig. 2). The tree includes established cohesin sequences, some of which were previously investigated experimentally in strain FD-1 (i.e., ScaA, ScaB, ScaC and ScaE) as well as a variety of putative cohesins (see Table S1). Many of the latter cohesins are found only in strain FD-1 (e.g., ScaJ, ScaK, ScaL, ScaM, ScaO and ScaP) as well as additional ORFs present in all three strains. Whether or not these protein modules constitute authentic cohesins remains an open question to be solved experimentally in the future.

The cohesins of the scaffoldins expressed by the different genes of the *sca* gene cluster, i.e., *scaC, scaA, scaB* and *scaE* (according to their order on the genome) are in general conserved among the strains according to previous findings ([57]). Thus, the ScaA cohesins of the three strains all appeared on the same branch. As anticipated, the first four ScaB cohesins of the FD-1 strain also co-clustered with the ScaA cohesins. The other ScaB cohesins (i.e., the last five ScaB cohesins of the FD-1 strain and all of the cohesins from strains 17 and 007c) co-clustered on a separate branch. Similarly, the ScaE cohesins co-cluster on a separate branch of the phylogenetic tree.

Many of the analogous scaffoldin sequences of strains 17 and 007c are remarkably similar and generally differ from their counterparts in strain FD-1. These include the cohesins of ScaG and ScaI as well as the cohesin sequence homologues of ScaC, ScaA, ScaB and ScaE. In contrast, the protein sequences of the ScaF cohesin are identical in all three strains. In addition, strains 17 and 007c contain an additional ScaF-like cohesin that differs somewhat from the ScaF cohesin. Strain FD-1 lacks the second ScaF-like cohesin.

Intriguingly, despite the near identity among most of the homologous cohesins of strains 17 and 007c, the ScaC cohesin in all three *R. flavefaciens* strains are conspicuously different in their sequences, thus reinforcing the notion that they may be used as a marker of the parent strain.

### Exceptional features of *R. flavefaciens* dockerins

We identified an unusually large and diverse pool of dockerin-containing proteins in all *R. flavefaciens* strains, compared with other cellulosome-containing species of Clostridiales, which ranges between 180 and 223 proteins (Table 1; 223, 180 and 183 dockerin-containing proteins in strains FD-1, 17 and 007c, respectively). These proteins bear a signal peptide, suggesting that they are secreted from the bacterium, and are often composed of cellulose-degrading catalytic modules as well as putative proteases, serpins, leucine-rich repeats and other unknown conserved protein modules as described earlier for strain FD-1 [34].We extensively explored the sequence conservation of each dockerin-containing protein, and identified its catalytic modules according to the CAZy database (see Materials and Methods). We profiled all modules of known GHs, PLs and CEs and classified them into family types, for both dockerin-containing proteins (Table 2) and other non-cellulosomal proteins (Table 3). Another group of dockerin-containing proteins contain non-catalytic modules, such as CBMs and domains of unknown function [34]. Of note are the catalytic modules that are unique to *R. flavefaciens* and absent in *R. albus*, such as GH families 18, 24, 42 and 97; CE families 13 and 15; and CBM families 32 and 63.

**Table 2:** Comparison of dockerin-containing CAZyme modules and CBMs of six Ruminococcaceae strains (in cellulosomal and non-cellulosomal proteins).

Glycoside Hydrolase																																									
	2	3	4	5	8	9	10	11	13	16	18	23	24	25	26	27	28	30	31	32	36	39	42	43	44	48	51	53	67	73	74	77	94	95	97	98	105	113	124	130	Total
***R. flavefaciens FD-1***	2	6	0	12	0	12	6	11	4	5	1	0	1	9	7	0	0	3	1	0	1	0	1	10	2	1	0	1	0	0	1	1	2	1	3	0	1	0	1	1	107
***R. flavefaciens 17***	1	4	0	9	1	14	4	18	4	5	0	0	0	5	4	0	0	2	1	0	1	0	1	7	1	1	0	1	0	2	1	1	2	2	1	0	1	0	1	1	96
***R. flavefaciens 007c***	1	4	0	9	1	13	4	17	4	5	0	0	0	6	4	0	0	2	1	0	1	0	1	7	1	1	0	1	0	2	1	1	2	2	1	0	1	0	1	1	95
***R. albus 7***	3	5	1	13	1	8	5	5	5	2	0	1	0	5	8	2	1	2	1	0	3	1	0	7	1	1	1	1	1	1	2	1	2	1	0	1	1	1	1	2	97
***R. albus 8***	4	6	1	14	1	7	3	3	5	1	0	1	0	5	6	1	1	1	1	0	2	0	0	9	1	1	1	1	1	1	2	1	2	2	0	0	2	1	0	2	90
***R. albus SY3***	3	5	0	12	1	12	8	7	6	2	0	1	0	6	4	2	0	4	1	1	3	1	0	7	1	1	2	1	1	1	1	2	3	1	0	1	0	1	1	1	104

Polysaccharide Lyase																																									
	1	9	10	11	Total																																				
***R. flavefaciens FD-1***	6	1	0	10	17																																				
***R. flavefaciens 17***	3	0	0	1	4																																				
***R. flavefaciens 007c***	3	0	0	1	4																																				
***R. albus 7***	3	1	1	2	7																																				
***R. albus 8***	2	1	1	2	6																																				
***R. albus SY3***	2	0	1	1	4																																				

Carbohydrate Esterase																																									
	1	2	3	4	8	9	12	13	15	Total																															
***R. flavefaciens FD-1***	7	3	6	6	1	0	5	1	1	30																															
***R. flavefaciens 17***	9	1	4	4	1	0	2	0	2	23																															
***R. flavefaciens 007c***	9	1	4	4	1	0	2	0	2	23																															
***R. albus 7***	3	3	1	4	2	1	4	0	0	18																															
***R. albus 8***	1	3	1	5	3	1	4	0	0	18																															
***R. albus SY3***	4	3	0	4	1	1	3	0	0	16																															

Carbohydrate-Binding Module																																									
	2	3	4	6	13	22	32	35	37	48	62	63	Total																												
***R. flavefaciens FD-1***	0	5	7	3	10	19	0	13	0	2	3	1	63																												
***R. flavefaciens 17***	0	5	8	6	4	16	2	6	0	2	2	1	52																												
***R. flavefaciens 007c***	0	5	7	6	4	16	2	6	0	2	2	1	51																												
***R. albus 7***	2	3	6	3	11	12	0	9	77	4	2	0	129																												
***R. albus 8***	1	3	5	2	8	7	0	7	51	4	0	0	88																												
***R. albus SY3***	1	4	8	4	4	17	0	6	102	5	4	0	155																												

Known families of each enzyme are marked in the header rows.

**Table 3:** Distribution of GH modules by families, with enzymes in indicated families containing either dockerins and/or CBM37.

	*R. flavefaciens*			*R. albus*					
	FD-1	*17*	007c	7		8		SY3	
	Dockerins			Dockerin	CBM37	Dockerin	CBM37	Dockerin	CBM37
**GH proteins shared by both species**									
**GH2**	1	1	1						1
**GH3**	1					1			
**GH5**	9	6	6	3	3	4	3	2	5
**GH9**	9	9	9	2	5	2	1		9
**GH10**	6	3	3		3	1*	1*		3
**GH11**	9 (10)†	9 (11)	7 (9)		4		2		3
**GH26**	3 (4)	3	3	3	2	2	1	1	1
**GH30**	3	2	2		2	1		1	3
**GH43**	7 (8)	6 (7)	6 (7)	3	1	3	1	4	1
**GH48**	1	1	1		1		1		1
**GH53**	1	1	1	1		1			1
**GH74**	1	1	1		1		1		1
**GH124**	1	1	1	1				1	
**GH proteins unique to ** ***R. flavefaciens***									
**GH8**		1	1						
**GH16**	1 (3)	1							
**GH44**	2	1	1						
**GH95**		1	1						
**GH97**	2	1	1						
**GH114**		1	1						
**GH proteins unique to ** ***R. albus***									
**GH25**								1	
**GH73**				1		1		1	
**GH98**					1				1
**GH105**						1			
**Total GHs**	107	96	95	97		90		104	
**Total GHs with dockerins**	50	49	46	14		17		11	
**Total GHs with CBM37s**				23		11		30	

† Numbers in parenthesis indicate the number of GH modules in enzymes which contain multiple GH modules of the same family.

^*^ One GH10 protein contains both a dockerin and a CBM37 module.

Table 4 describes a group of dockerin-containing enzymes that contains more than one type of catalytic module on the same polypeptide chain. *R. flavefaciens* codes for a relatively large number of such “multifunctional enzymes”. One of the dominant modules is GH43, which has been recently shown to be abundant in the rumen in metagenomic studies [59], [60] and is one of the more abundant GH enzyme families in the genomes of common hemicellulolyic rumen bacteria [61], [62]. The GH43 family exhibits broad substrate specificity and promiscuous characteristics [61], [63]. It is clear that strains 17 and 007c share numerous protein architectures, many of which are different from those of strain FD-1. This observation may indeed reflect the relatedness between strains 17 and 007c and their distinction from strain FD-1.

**Table 4:** Cellulosomal and non-cellulosomal multifunctional proteins in *R. flavefaciens*.

Domain architecture (cellulosome-related domains)	*R. flavefaciens* accession numbers		
Strain	FD-1	17	007c
**Shared by all strains:**			
CBM13-*Doc*-**GH43**-**GH43**	ZP_06142338	WP_019678907	orf03036
CBM22-**GH10**-CBM22-*Doc*-**GH43**-CBM6	orf03865	WP_019680029	EWM52826
**CE12**-CBM13-*Doc*-CBM35-**CE**12	orf02983, orf03219	WP_019678069	EWM54325
**CE8**-**PL1**-*Doc*	orf02371	WP_00998568	EWM52494
**GH11**-CBM22-**GH10**-*Doc*-CBM22-**CE4**	orf01222	WP_019679223	EWM54891
**GH25**-**GH25**	ZP_06141601	WP_019678757	EWM53404
**GH43**-CBM22-*Doc*-**CE1**	orf00341	WP_009983072	EWM54432
**GH43**-CBM6-CBM22-*Doc*-**CE1**	orf00764	WP_019678371	EWM53765
			
Shared by two strains:			
CBM22-*Doc*-**CE1**-**CE1**		WP_019678253	EWM55310
**CE3-** *Doc*-**CE15**		WP_019679655, CAB55348	EWM52579, EWM54090
**GH11**-CBM22-*Doc*-**GH16**		AAB26620 (reported in [82])	EWM53768
**GH11**-**CE1**		orf01851	orf04775
**GH11**-**CE4**		orf02455	orf00919
**GH11-GH10**		P29126 (reported in [83])	orf01418
**GH11**-**GH11**-*Doc*		WP_019679180	EWM54934
**GH9**-**GH16**		orf02516	orf00858
			
Strain specific:			
CBM35**-CE3**- *Doc*-**CBM35-GH26**	orf03447		
CBM35**-CE3**-**GH5**-*Doc*	orf00227		
**CE3**-CBM22-*Doc*-**CE15**	orf02390		
*Doc*-**GH16**-**GH16**-**GH16**	orf00265		
**GH11**-CBM13-**CE**1- *Doc*	orf00775		
**GH11**-CBM22-*Doc* **-GH11**-**CE1**	orf03180		
**GH11**-CBM22-*Doc*-**GH11**-**CE3**	orf01315		
**GH11**-CBM22-**GH10**- *Doc* **-GH11**	orf00468		
**GH11**-CBM22-**GH10**- *Doc*-**GH11**-**CE4**	orf03896		
**GH11**-**CE3**-*Doc*	orf01321		
**GH53-CE3-** *Doc*	orf01739		
**GH5-GH5**-*Doc*	orf01388		
**PL1-PL9**-X215-*Doc*	orf00696		
**PL11**- *Doc*-CBM35-**CE12**	orf03451		
**GH11**-**GH16**		orf01699	
**GH11**-CBM22-**CE3**- *Doc*		CAB93667 (reported in [84])	

Compared with other rumen bacteria we noted a group of exclusive enzymes, which are unique to the *R. flavefaciens* strains and are absent or underrepresented in the genomes of *R. albus* strains and other fibrolytic rumen species, e.g., *Fibrobacter succinogenes* subsp. succinogenes S85. These include β-galactosidases (GH42), α-glucosidases (GH97), xylanases (GH11) and proteins with an unusual number of PLs from family 11 (Table 2).

The conserved sequence pattern of *R. flavefaciens* FD-1 dockerins was examined previously [33], [34], and the data supported the classification of all dockerins in that genome into six major groups. Subtypes of dockerins with unique features were described, that included atypical lengths of the second calcium-binding repeat, different sequence insertions and different linkers within the dockerin module. When comparing dockerins from the three *R. flavefaciens* strains we observed a similar trend of diversity and heterogeneity in the sequences of dockerins (Fig. S1). Interestingly, there are only three identical dockerins between strain FD-1 dockerins and those of strain 17 or 007c. Strain FD-1 dockerins are on average 46% similar to homologues in 007c and 67% similar to those of strain 17. BLAST searches with dockerin members from FD-1 groups as queries revealed homologous dockerins (e-value <10^−10^) in strains 17 and 007c, except for group 4 b dockerins which were exclusive to strain FD-1.

Overall, we identified genes coding for an elaborate and sophisticated cellulosome in all three *R. flavefaciens* strains. Notably, we observed particular variations in the composition and in the number of key cellulosomal elements between the different strains. Of the major novel architectures is a multi-dockerin protein (EWM52407 in *R. flavefaciens* 007c and WP_019680459 in *R. flavefaciens* 17), which contains seven tandem non-identical dockerin repeats and appears in strains 007c and 17 but not FD-1. This novel protein architecture has yet to be observed in any other cellulosome-producing bacterium. In addition, another rare protein arrangement of two non-tandem repeats of a dockerin in the same polypeptide was observed in these strains (EWM52383 in *R. flavefaciens* 007c and orf03158 in *R. flavefaciens* 17), and joins a recent observation of this type of protein in *Acetivibrio cellulolyticus* [64].

### *R. albus* is cohesin-deficient yet encodes for dockerins and cell-anchoring modules

In order to further understand the cellulosomics of *R. albus*, we sequenced the genome of *R. albus* SY3 and compared it to the two publicly available genomes of *R. albus*, strains 7 and 8 (Fig. 1B and Table 1). Genome-wide analysis of the three *R. albus* strains revealed 90, 62 and 58 dockerin-containing proteins in strains 7, 8 and SY3, respectively. Unlike *R. flavefaciens*, these dockerins are generally conserved and could not be divided into significant subgroups. The predominant predicted recognition residues in all three *R. albus* strains were V(I), T, A and A in positions 10, 11, 17 and 18 of the repeated segment.

Surprisingly, only one cohesin-containing protein was determined in the genomes of *R. albus* strains 7 and SY3, and none in strain 8 (GI number 317056975 and EXM40378, respectively). The single cohesin module is supplemented by a C-terminal dockerin module and a linker between the two, thus resembling an “adaptor” cohesin-dockerin protein, similar to that of ScaC in *R. flavefaciens*. The two homologous *R. albus* cohesin-containing proteins are 92% similar. Comparison of the cohesin module with *R. flavefaciens* cohesins showed 69% similarity (with *R. flavefaciens* 17) and 79% (with *R. flavefaciens* FD-1). This single *R. albus* cohesin is orthologous to the *R. flavefaciens* ScaF protein (Fig. 2). The apparent presence of a lone cohesin in *R. albus* represents a puzzling deviation from the classical cellulosome architecture, where dockerins are anchored onto multiple cohesin-containing scaffoldins. These observations suggest an alternative mechanism for immobilization of dockerin-containing enzymes onto carbohydrates or their anchoring to the cell surface.

*R. albus* contains CBMs belonging to several family types (Table 2), two of which (family 2 and 37) are absent in *R. flavefaciens*. The cellulose-binding CBM2 (common in numerous non-cellulosomal cellulolytic bacteria) appears in only one or two copies in proteins that also contain a GH5 module. More intriguingly, all three *R. albus* genomes contain multiple copies of a family 37 sugar-binding module (CBM37), which is unique to this species (77, 51 and 102 copies in *R. albus* 7, 8 and SY3, respectively). The CBM37 module is absent in *R. flavefaciens*, and has not been detected in any other sequenced genome. This special CBM is integrated into various carbohydrate-active proteins, in association with catalytic modules such as GHs, CEs, as well as non-catalytic proteins, but very rarely with dockerins – only observed once per strain. In several cases in all three organisms, the CBM37 module appears in a tandem repeat (13, 11, and 18 in strains 7, 8 and SY3, respectively).

We examined the co-appearance of two modules, CBM37 and GHs, in the same protein (Table 3). CBM37 was associated with 11 different GH families, including cellulases (GH5, GH9, GH48) and hemicellulases (GH5, GH10, GH11, GH26, GH43). Interestingly, some of the GH families appear both in *R. flavefaciens* and in *R. albus*, the latter of which are also associated with CBM37 (with one exception, GH98).

The distribution of GH modules within the dockerin-containing enzymes (Table 2) shows that *R. albus* codes for modules from unique GH families, which are exclusive to that species, such as family 4 (acetyl xylan esterase), family 23, family 27, family 28 (polygalacturonase), family 32, family 39 (α-L-iduronidase and β-xylosidase), family 51 (endoglucanase/endoxylanase), family 67 (glucuronidase), family 98 (endo-β-galactosidase) and family 113 (β-mannanase). The *R. albus* genome also codes for PL10 and CE9 modules, which are absent in *R. flavefaciens*.

*R. albus* codes for 4–8 multifunctional proteins (Table 5), some of which have a common protein architecture in two of the strains, while others are strain-specific. Five of these proteins contain GH11-CBM22 modules, with a different C-terminal variation on the protein. Strain 7 and SY3 share more multifunctional protein architectures with each other than with strain 8. The number of multifunctional proteins in *R. albus* is significantly less than those of *R. flavefaciens*.

**Table 5:** Cellulosomal and non-cellulosomal multifunctional proteins in *R. albus*.

Domain architecture (cellulosome-related domains)	*R. albus a*ccession numbers		
Strain	7	8	SY3
**Shared by two strains:**			
CBM35-**GH26**-**CE3**-***CBM37***	YP_004103508	ZP_08158982	
**CE12**-CBM13-***Doc***-CBM35-**CE12**	YP_004103674		EXM39991
**GH11**-CBM22-***CBM37***-**CE1**	YP_004105842		EXM39976
**GH11**-CBM22-***CBM37***-**CE4**	YP_004104068		
**GH11**-CBM22-**CE4**	YP_004103272		EXM39050
**GH11**-CBM22-**GH10-** ***CBM37***	YP_004090078		EXM37450
**GH43**-CBM22-CBM22-***Doc***-**CE1**	YP_004104621		EXM37569
**PL1**-**PL1**-***CBM37***	YP_004105710		EXM39993
**Strain specific:**			
**CE12**-CBM13-***Doc***-CBM35-**CE**12		ZP_08160451	
**PL10**-**CE8**-***Doc***		ZP_08159991	
**PL11**-**CE12**-CBM13-CBM13-***CBM37***		ZP_08159623	
**PL10**-**CE8**-***CBM37***			EXM38121

Protein domain architecture is described, including only cellulosome-related domains.

## Discussion

The microbial community of the rumen shares a rich source of novel plant cell wall degrading enzymes, which include cellulases, xylanases and other hemicellulases, as well as pectinases [65]. Although cellulolytic enzyme systems have been investigated over the years, the mechanisms by which bacteria achieve efficient plant cell wall breakdown are still obscure. In this work we have described a multi-dimensional perspective on the cellulolytic potential of the two dominant fibrolytic ruminococci, *R. flavefaciens* and *R. albus* by comparing the cellulase system of three different strains from each species. Divergent mechanisms of fiber degradation were revealed by integrating the data, which involved (i) the outlining of their scaffoldins and dockerin-containing proteins, (ii) the profiling of cellulose-degrading enzymes in each species and strain, and (iii) the identification of protein architectures of complex multifunctional enzymes of each strain.

All *R. flavefaciens* strains code for particularly elaborate cellulosome systems, having multiple cohesin-containing proteins that may assemble into defined cellulosomal structures, which exhibit various combinations of dockerin-containing cellulases on their surface. Distinct differences in the number of enzymes (Table 2) or their modular architectures (Table 4) were observed among the different *R. flavefaciens* strains. Based on these observations it is likely strains 17 and 007c are more closely related to one another than either is to FD-1. This is also reflected by the phylogenetic relatedness of the cohesin sequences of the former two strains versus those of the latter. It is also clear that strain FD-1 bears the most elaborate cellulosome system. Sequence variability in the structural *sca* gene cluster (*scaC-scaA-scaB-cttA-scaE*) was also supported by a previous work [53], suggesting that other *R. flavefaciens* strains may reflect such strain-related plasticity. Indeed, recent work, which explored the diversity of *R. flavefaciens* strains in the rumen using the polymorphic nature of ScaC [52], revealed spatial and temporal differences among strains that may relate to functional differences among *R. flavefaciens* strains.

Analysis of the cellulolytic gene complement of *R. albus* raises questions regarding its approach to degrade cellulose fibers. Each genome contains several dozens of dockerins. Surprisingly, however, only a single cohesin-containing protein was detected in strains 7 and SY3, and a cohesin counterpart was not detected in strain 8. These findings do not coincide with the classical cellulosome paradigm, whereby multiple cohesin-bearing scaffoldins are essential for enzyme assembly, and it is thus difficult to assign a functional role for the dozens of dockerins that are conserved in the *R. albus* genomes. Indeed, a broad range of non-cellulolytic microbes that lack appropriate GH and other CAZymes have been found to possess numerous genes encoding dockerin-containing proteins, and in many cases genes for cohesins are either lacking or appear in only a single copy [66]. This clearly implies that the latter microbes (mainly bacteria and archaea) do not produce *bona fide* cellulosome-like structures, which raises the question as to what is the exact role of the dockerin in these proteins. It was previously suggested that such dockerins may bind an as-yet undetermined protein component or they may be involved in other reactions [66]. Nevertheless, in *R. albus* many of the dockerins are borne by CAZymes, and the rich rumen ecosystem may provide appropriate scaffoldins in an interspecies manner (e.g., those of *R. flavefaciens*) that may accept them symbiotically. Thus, an alternative mechanism might involve a collaborative usage of cohesins and dockerins of both *R. flavefaciens* and *R. albus* for putative hybrid cellulosomes where *R. flavefaciens* cohesins would incorporate both its own dockerin-bearing components and those of *R. albus*. Interestingly, some dockerin-containing proteins in *R. albus* are encoded by plasmid genes (e.g. in strain 7, two plasmid, pRUMAL01 and pRUMAL02 encode nine such proteins). It is thus possible that the ruminal microbial communities adjust to environmental changes by sharing and acquisition of advantageous components, such as dockerin-containing proteins, via interspecies exchange of plasmids [67].

Despite the lack of a genuine cellulosome, *R. albus* is known to degrade cellulosic substrates to levels similar to those of *R. flavefaciens* [68]. In this context, our analyses highlight a key role for a dominant and unique protein module in *R. albus*, CBM37, that appears to provide an alternative strategy for this bacterium. CBM37s appear in high copy number in all three *R. albus* strains, and their numbers vary greatly among them. Indeed, this particular module has been shown definitively to attach enzymes directly to bacterial cell wall carbohydrates [51]. Interestingly, CBM37s are distributed in many *R. albus* enzymes whose orthologs in *R. flavefaciens* are instead equipped with dockerins. Notably, the critically important family 48 cellulase bears a CBM37 in all three *R. albus* strains, as does the family 74 xyloglucanase and the family 11 xylanases. This observation raises the intriguing possibility that CBM37 is the major mechanism for cell-surface anchoring of the cellulolytic and associated enzymes instead of the classical type of scaffoldin that positions them in close proximity to the bacterial cell. Of note is the disproportionate number of dockerins and CBM37s in strain SY3 versus the other two strains, mainly due to a higher copy number of GHs with CBM37 modules (Table 1).

The rumen microbial population is dynamic and complex in terms of its biodiversity, exhibiting both competitive and symbiotic types of relationship [69]. The conditions in the rumen may thus allow the variety of *R. flavefaciens* strains to share substrates as well as promote cross-strain symbiosis, whereby the strains can share cellulosomal components and/or benefit together from their degraded products. Thus, closely related strains of *R. flavefaciens* have homologous dockerin and cohesin components, which raises the hypothesis that such structural components and enzymes may be interchangeable when secreted. This may expand the number of combinations for building a cellulosome and increase its diversity. In spite of the benefits that may be derived from the exchange of components, there is evidence for competition in the utilization of either cellulose or cellobiose in co-cultures of *R. albus* and *R. flavefaciens* [70]. The nature of the catalytic enzyme may be another tool employed by the bacterium for a competitive advantage and efficient cellulose degradation. Both *R. flavefaciens* and *R. albus* code for various carbohydrate-degrading enzymes, yet each species also codes for exclusive families of GHs, PLs and CEs (Table 2). This trend is also reflected in the arrangement of the multifunctional proteins, which are very abundant in *R. flavefaciens* compared to other known Firmicutes, and compared to *R. albus*.

An additional species dominant in the fibrolytic consortium of the rumen is *Fibrobacter succinogenes*. Its genome does not code for known cellulosomal components, yet it codes for over a hundred predicted carbohydrate-active enzymes [71], exhibiting catalytic activities of cellulases, xylanases, PLs and CEs. A comparison of the enzymatic profile between this genome and all six ruminal genomes shows that *F. succinogenes* exclusively codes for GH families which neither appear in *R. flavefaciens* nor *R. albus*, such as family 45 (endoglucanases), family 54 (α-L-arabinofuranosidases and β-xylosidases), family 57 (α-amylases and others) and family 116 (β-glucosidases and β-xylosidases). Interestingly, endocellulases from GH family 45 are rare in bacteria, and are more common in eukarya. *F. succinogenes* also contains PL family14 and CE family 6, which are absent in the ruminococci. Of note is the unique profile of CBMs in the *F. succinogenes* genome. The presence of family 6 CBMs is expanded in its genome to 25 copies, while CBMs important for crystalline cellulose degradation (families 2 and 3) are absent. Most of its CBMs (5 types out of 7) belong to families which are absent in *R. flavefaciens* and *R. albus* genomes. One possible mechanism for *F. succinogenes* fiber degradation has been suggested by Brumm et al [71], who proposed a molecular “motor” which removes glucan chains from cellulose crystals and transports them, using energy derived from cellulolysis.

The present work surveys the different strategies by which two ruminococcal species can degrade cellulose fibers, by analyzing the encoded cellulosomal and enzymatic proteins from their genomes. The extreme diversity of enzymes and structural scaffoldins was demonstrated within *R. flavefaciens* and *R. albus* strains, and also between these species. It is yet to be understood how the elaborate arsenal of CAZymes and the different cohesin-containing components are being regulated in the rumen. This work highlights the need for more extensive experimental studies to assess the spatial and temporal organization of the multiple cohesins, dockerins and enzyme activities of these species in the rumen.

## Materials and Methods

### Genome sources

Six genomes were explored in this work, three strains each of *Ruminococcus flavefaciens* (FD-1, 17 and 007c) and *Ruminococcus albus* (7, 8 and SY3) (Table 6). *R. flavefaciens* FD-1 was isolated by M. Bryant from a pill containing ruminal organisms in 1953 in Maryland, US [1] and *R. flavefaciens* 17 was isolated from the rumen of a Friesian cow that received a diet of grass cubes, hay, and concentrates at the Rowett Institute in Aberdeen, UK [72]. *R. flavefaciens* 007c is another Rowett strain isolated from rumen contents of a cannulated cow that was fed hay and starchy concentrates, and shares with strain 17 the ability to degrade dewaxed cotton cellulose [73], [74]. *R. albus* SY3 was also isolated at the Rowett, in 1976 [74]. *R. albus* 7 (a type strain, ATCC 27210, DSM 20455) was isolated in 1951 by M. Bryant from a Holstein cow fed alfalfa hay-grain [1]; *R. albus* 8 is an isolate from the rumen of an alfalfa hay-fed cow [75]. The genomes of *R. albus* 8 and *F. succinogenes* S85 were sequenced by the North American Consortium for Rumen Bacteria at The Institute for Genome Research (now the J. C. Venter Institute). Standard methods used at TIGR during this period for library construction, DNA sequencing (Sanger-based technologies) and data assembly were employed [62].

**Table 6:** Summary of genomes analyzed in this work.

Cellulosome-producing bacteria	Genome source	Assembly Draft	GenBank ID	Number of contigs	Number of predicted proteins	Total nucleotides	Reference
*R. flavefaciens* FD-1	The North American Consortium for Genomics of Fibrolytic Ruminal Bacteria	25-AUG-2009	ACOK00000000	119	3878	4,573,608	[33]
*R. flavefaciens* 17	University of Illinois	10-FEB-2012	AFNE00000000	489	3192	3,454,940	[85]
*R. flavefaciens* 007c	Wellcome Trust Sanger Institute	11-Dec-2009	ATAX01000000	39	3,185	3,649,758	This work, [77]
*R. albus* 7	JGI-PGF	26-JAN-2012	ASM17963v2	Gapless Chromosome	3,872	4,482,087	[86]
*R. albus* 8	JCVI	25-FEB-2011	ASM17815v2	136	3,833	4,052,160	[75]
*R. albus* SY3	University of Illinois	29-May-2013	RaSY3	81	3,654	3,832,777	This work

### Genome sequencing of *R. albus* SY3

*R. albus* SY3 was sequenced at the W.M. Keck Center for Comparative and Functional Genomics (University of Illinois at Urbana-Champaign). Total sequence data was generated from both a paired-ended 500-nt insert library sequenced on a single lane of HiSeq (Illumina) and a paired ended 3-kb insert library sequenced on a full plate of 454 sequencing (Roche Diagnostics). These approaches yielded 47 million 100-nt reads (4.7 billion bases) and 1.4 million reads with an average read length of 402 nt (577 million bases; 71% true paired end, actual paired distance was 2386+597 nt), respectively. The 454 sequence data was assembled using Newbler v2.5.3 and the Illumina was assembled using Velvet v1.1. The assemblies were combined using Minimus2. The sequence assembled to 4 scaffolds (N50 = 1,120,630 bp) and 97 contigs (N50 = 114,193). 99.95% of bases were >Q40 and all others (1808 bp) were Q39. The total sequence produced was 3,832,777 nt and the genome was estimated to be 4.1 Mb, giving us 93.5% coverage. The modal sequence coverage depth was 131×. The sequence was annotated using subsystems in RAST.

### Genome sequencing of *R. flavefaciens* 007c

Genome sequencing of strain 007c was performed at the Wellcome Trust Sanger Institute, Cambridge UK, courtesy of Keith and Julian Parkhill, based on 454 pyrosequencing, with paired-end reads. *Ruminococcus flavefaciens* 007 was isolated from rumen contents of a cannulated cow that was fed hay and starchy concentrate, at the Rowett Institute in Scotland, as reported by Stewart CS et al (1981) [76]. This was the only one of 54 single colony isolates selected by their ability to form clear zones in cellulose agar roll tubes (all reported to be ruminococci) that was able to cause significant weight loss from dewaxed cotton fiber. Thus it is one of the most active *Ruminococcus* strains to have been isolated with respect to this highly recalcitrant form of cellulose. This paper reported 78.1% weight loss from cotton fiber within 7 days for *R. flavefaciens* 007, compared with 81.4% for *Fibrobacter succinogenes* BL2 (which was the most active *Fibrobacter* strain isolated). *Fibrobacter* strains do not form clear zones in cellulose agar, but were isolated from enrichment cultures. Subsequently, subcultivation on medium containing cellobiose but no cellulose was found to result in a loss of cotton-degrading activity by 007, but this activity could be regained by serial subculture on cotton. The derivative strains retaining, or lacking, cotton-degrading activity were referred to as 007c and 007s, respectively [73]. The proteomes of these two strains have been compared recently and exhibit some potentially key differences [77]. This Whole Genome Shotgun project has been deposited at GenBank under the accession ATAX00000000. The version described in this paper is version ATAX01000000.

### Sequence identification of cohesins and dockerins

A genome-wide survey was conducted to predict cohesion- and dockerin-containing proteins. Proteins were subjected to BLAST [78] searches, using sequences of known cohesin and dockerin modules as queries. Retrieved hits below E-value of 10^−4^ were individually inspected by examining their characteristic sequence features and protein architecture. Obvious dockerin modules were expected to contain two Ca^+2^-binding repeats, putative helices and linker regions. Low-scoring hits of dockerins and cohesins were examined by comparing them against known dockerin or cohesin sequences, respectively. Multiple sequence alignments were obtained using CLUSTALW [79], with manual corrections when needed. The cohesin dendrogram was generated using PhyML algorithms (with LG substitution model, and default parameters of the Approximate Likelihood-Ratio test) [80] and visualized using TreeView [81].

### Annotation of CAZymes

Both cellulosomal and non-cellulosomal proteins were annotated by the CAZy pipeline (http://www.cazy.org) [54], in order to predict their catalytic modules. This includes identification of the catalytic modules and their classification into family types, according to sequence conservation, for glycoside hydrolases, carbohydrate esterases, polysaccharide lyases, carbohydrate-binding modules and glycosyl transferases. Additional conserved domains of the proteins were analyzed using the CD-search website (http://www.ncbi.nlm.nih.gov/Structure/cdd/wrpsb.cgi) and the Pfam database (http://pfam.sanger.ac.uk/).

## Supporting Information

Figure S1 Alignments of homologous *R. flavefaciens* dockerins.
(PDF)

Table S1 Protein architectures of identified scaffoldins.
(PDF)
