# Supplementary material for: Rumen Cellulosomics: Divergent Fiber-Degrading Strategies Revealed by Comparative Genome-Wide Analysis of Six Ruminococcal Strains
Source: PLoS One. 2014 Jul 3;9(7):e99221. doi: 10.1371/journal.pone.0099221 (PMC4081043; doi:10.1371/journal.pone.0099221)
Supplement: Figure S1 — Alignments of homologous R. flavefaciens dockerins. (PDF) [file pone.0099221.s001.pdf]

Supporting Information S2: Alignments of homologous *R. flavefaciens* dockerins

Representative sequences of dockerins from *R. flavefaciens* strains FD-1, 17 and 007c are aligned according to the major subgroups as previously reported for *R. flavefaciens* FD-1 dockerins (Rincon MT, Dassa B, Flint HJ, Travis AR, Jindou S, et al. (2010) Abundance and diversity of dockerin-containing proteins in the fiber-degrading rumen bacterium, *Ruminococcus flavefaciens* FD1. PLoS ONE 5: e12476.).

Color-shading reflects a minimum sequence conservation of 70% similarity, and sequence titles include the strain name and its ORF number.

Group 1(a)

```
FD-1_00059     GDANCDGVVDISDAVIIMQSISNPSKYKLTEEGRKNADVTGKNDGVTNNDALAIQKLMLKLIDKLPEE
FD-1_00060     GDANCDGVVDISDAVIIMQSISNPSKYKLTEEGRKNADVTGKNDGVTNNDALAIQKLMLKLIDKLPEE
FD-1_00389     GDANCDGKVDLSDAVRIMQAISNPSKYKLNDEESANADCSGGNDGVTNGDALAIQKYCLSLITSLPEE
17_02566       GDANCDGTVDLADAIFIMQALANPNKYKLSETGRANADVCEAGGGVTNDDAVTIQRKLIGLVSTLPES
17_03291       GDVNGDGVVELADAILIMQSLANPDKYKIAPELRTNADVEQRGNGITGSDAVAIQKFLIGLIESLPEV
17_02094       GDANGDGVVDLADAIFIMQCLANPNKYQLSDAGRVAADVYG-DDGVTGDDAMAIQLLLIKKISSLPVP
007c_00805     GDANCDGTVDLADAIFIMQALANPNKYKLSETGRANADVCEAGGGVTNDDAVTIQRKLIGLVSTLPES
007c_0007c6    GDVNGDGVVELADAILIMQSLANPDKYKIAPELRTNADVEQRGNGITGSDAVAIQKFLIGLIESLPEV
007c_03086     GDANGDGVVDLADAIFIMQCLANPNKYQLSDAGRVAADVYG-DDGVTGDDAMAIQLLLIKKISSLPVP
```

Group 2

```
FD-1_01750     GDINGDGIIDGRDATVLLTYYA-KTSTGYKGSLMKFMEEQNIIQGDDI--------YSGTFRL
FD-1_02067     GDIDGNDMIDGRDASILLTYYA-KTSTGYTGTLEEYVESVKETESSGS--------LMDFFSE
FD-1_03182     GDINNDTIIDGRDASILLTYYA-KTSTGYKGNLQSFVGEQ---IGIDP--------LTGQTVI
17_04856       GDVNNDSIVDSVDASLILAEYA-LTSTGKTGSFNASQSKCADVDRSGTVDSVDASKVLAYYSY
17_00511       GDVNNDGMIDAVDASVVLAYYA-RISTNHDGGFTELQKLAADFNHDGKVDAVDASNILSYYTY
17_04480       GDINSDGLVDAVDASGVLAEYAKLSSKNGKGDFTADQRKAADVDKNDKVDSVDASKILAYYAY
007c_04535     GDVNNDSIVDSVDASLILAEYA-LTSTGKTGSFNASQSKCADVDRSGTVDSVDASKVLAYYSY
007c_01979     GDVNNDGMIDAVDASVVLAYYA-RISTNHDGGFTELQKLAADFNHDGKVDAVDASNILSYYTY
007c_04621     GDVNNDRLINAVDASEVLTYYT-MTSTNKNGDLDANQKLAADVDRNGSINAVDASNILSYYAY
```

Group 3

```
FD-1_00341     GDINSDGCIDSFDVIAGRKGIING-LSGIAA---KKAADVDGNGSFQINDLILIHKFVLGEKKDF
FD-1_00381     GDVNFDGAVGLADAVQLNRYLLAE-NAELGN---WKNADIAEDGTIDVFDLIYLRRQLLGEKPPV
FD-1_00408     GDLNIDGVIDIFDLISMKKLILSG-TASASS---LAAADVNRDGDITIGDAVLIQRFIEGSVTDF
17_03157       GDINYDGVIDSFDIILARRGLIKG-ITDKKA---LKAADADSNGVFEINDALLIMQFVSGKIKEF
17_00852       GDVNFDECIDSLDMITARKGLIKGGFDDAMT---QKAADVDQNKTFEVADLVLLQQYVLKKITEF
17_01934       GDFDSDGYLTSFDLIGARRALLSH-MSGSDAGINMNIADIDGDGSFAVNDLVLLTKFLLGQVTEF
007c_04691     GDFDSDGYLTSFDLIGARRALLSH-MSGSDAGINMNIADIDGDGSFAVNDLVLLTKFLLGQVTEF
007c_00204     GDINYDGVIDSFDIILARRGLIKG-ITDKKA---LKAADADSNGVFEINDALLIMQFVSGKIKEF
007c_03040     GDVNFDGVIDSLDMIAARKAIIKGDLTGAS----LKAADVDQNGKFEVADLVNIKKFILRLITEW
```

Group 4(a)

```
FD-1_00081     GDVNNDMIVDARDASDILTGYAMMSVGDD----SDLDPVLADYDFNGHIDAIDASKVLTDYAKSSA
FD-1_00251     GDVDGDASVTAKDATAVLTEYAKTSVGKEG-SFSDRAKKSADVDRNGKIDGVDASTILSFYAYSSV
FD-1_00270     GDANLDGIIDAVDATLVLTDYARVSVGKTT-TLNEAQRGNADMNLNGIIDAVDASAILTYYAKSSV
17_04623       GDVNRDGKADAVDASMVLAEYAAVTAHRSS-TLNQAQKARGDLNGDSKLDSVDASQILAYYAKNST
17_00275       GDVNFDNKVDSVDASLILAEYAKISTNQNT-TFNEKQFTAGDVDETNVIDSVDASKVLAYYSYTST
17_04856       GDVNNDSIVDSVDASLILAEYALTSTGKTG-SFNASQSKCADVDRSGTVDSVDASKVLAYYSYTST
007c_04621     GDVNNDRLINAVDASEVLTYYTMTSTNKNG-DLDANQKLAADVDRNGSINAVDASNILSYYAYTST
007c_01979     GDVNNDGMIDAVDASVVLAYYARISTNHDG-GFTELQKLAADFNHDGKVDAVDASNILSYYTYIST
```

```
007c_04937      GDVNADGNVDSIDSSFVLIEYSKLSSGNGIGSFNDEQRRAADVDKNGITNAVDASKILWYYAYSST
```

Group 6(a)

```
FD-1_00886      GDANADGVFDVADAVLLQKWLLAVPDMHLENWQAADLCKDNRLDVFDLCLMKRELI
FD-1_00903      GDVNASGKVDIADLVLLQKWLLSVPDTYLPNWKAGDLCQDEKLDVFDLCALRRLLL
FD-1_01307      GDLNSDGTFNVSDAVLLQKWLLSDYKTNLKNWKNGDFVEDNVLDIFDLCLMKKALI
17_01711        GDVNNDGEFNISDVVLLQKWLLTVPDTHLSNWKAADLCDDNRLDVFDLCLMRRKLI
17_00021        GDVNGDGEFSISDVVLLQKWLLAVPGTELADWKAADLCKDDELDVFDLCMMRKALI
17_01207        GDVNDDGEFGVADLVIFQKWLLADKDTKLKKWRAADLCRNNRLDVYDLVLMRKELL
007c_04976      GDVNNDGKFNISDVVLLQKWLLTVPDTHLSNWKAADLCDDNRLDVFDLCLMRRKLI
007c_04633      GDVNDDGEFNVADVVLLQKWLLADPDIHLSNWKAADFCNDNKLDVFDLCLMRRELL
007c_04650      GDINNDGEFNVADVVLLQKWLLAVPDTHLANWETADFCRDNNLDIFDLVLMRKALL
```
