# Supplementary material for: Rumen Cellulosomics: Divergent Fiber-Degrading Strategies Revealed by Comparative Genome-Wide Analysis of Six Ruminococcal Strains
Source: PLoS One. 2014 Jul 3;9(7):e99221. doi: 10.1371/journal.pone.0099221 (PMC4081043; doi:10.1371/journal.pone.0099221)
Supplement: Table S1 — Protein architectures of identified scaffoldins. (PDF) [file pone.0099221.s002.pdf]

Rumen cellulosomics: Divergent fiber-degrading strategies revealed by comparative genome-wide analysis of six ruminococcal strains

Bareket Dassa[1], Ilya Borovok[2], Vered Ruimy-Israeli[1], Raphael Lamed[2], Harry Flint[3], Sylvia Duncan[3], Bernard Henrissat[4], Pedro Coutinho[4], Mark Morrison[5,6], Pascale Mosoni[7], Carl J. Yeoman[8], Bryan White[9,10] and Edward A. Bayer[1]*

Supporting Information S1 Table: Protein architectures of identified scaffoldins

	Protein name and GenBank ID	N= Incomplete sequence	Comments
Ruminococcus flavefaciens 17	CAC34384 **ScaA**	CohX-Coh-Coh-N-Coh-Doc	Incomplete sequence
	CAC34385 **ScaB**	Coh-Coh-Coh-Coh-Coh-Coh-Coh-XDoc	
	CAE51046 **ScaC**	Coh-Doc	
	CAH18996 **ScaE**	Coh-sortase	
	WP_019679656 **ScaF**	*-Coh-Doc	SP recognized only by version 3 of SignalP
	orf02408	*Coh-N	Incomplete ORF, No SP, related to ScaA3; perhaps sequencing anomaly
	WP_019680574 **ScaF-like**	Coh-Doc	
	orf04875 **ScaI**	LRR-Coh-Doc	
	WP_019678998	Coh-Doc	
	WP_019680026 **ScaG**	Coh	
	WP_019678225	Coh?	
	WP_019678123	Coh/FN3-peptidase/ Sortase	
Ruminococcus flavefaciens 007c	EWM54564 **ScaA**	CohX-Coh-Coh-Coh-Doc	
	EWM54563 **ScaB**	Coh-Coh-Coh-Coh	Incomplete ORF; additional cohesin(s) and C-terminal XDoc assumed
	EWM54565 **ScaC**-like	Coh-Doc	
	EWM54693 **ScaE**	Coh-Sortase?	
	EWM52511 **ScaI**	Coh-Doc	
	EWM52578 **ScaF**	*-Coh-Doc	SP recognized only by version 3 of SignalP
	EWM52383 **ScaH**	**Doc-Coh-Doc-Doc?**	
	EWM52234 **ScaF-like**	Coh-Doc	
	EWM54679	Coh-Doc	

	EWM52829 **ScaG**	Coh	
Ruminococcus flavefaciens FD-1	268610846 **ScaA**	CohX-Coh-Coh-Doc	
	268610847 **ScaB**	Coh-Coh-Coh-Coh-Coh-Coh-Coh-Coh-Coh- XDoc	
	268610845 **ScaC**	Coh-Doc	
	268610849 **ScaE**	Coh-Sortase?	
	268608634 **ScaH**	Coh-Doc	
	268610861 **ScaF**	*-Coh-Doc	
	268608381 **ScaG**	Coh	
	268611770 **ScaJ**	Coh-Coh-Doc	
	268610808 **ScaO**	Coh-Doc	
	268610314 **ScaN**	Coh-Doc	
	268611807 **ScaK**	Coh-Doc	
	268609264 **ScaE**	Coh-Doc	
	268610316 **ScaP**	*Coh	NO SP
	268609943 **ScaL**	Coh-Doc	
	268611778 **ScaM**	Coh-Doc	
	268609953	Coh-Coh-Doc	
	268612017 **ScaI**	Coh	
Ruminococcus albus 7	317056975	Coh-Doc	
Ruminococcus albus SY3	EXM40378	Coh-Doc	
